# Genome sequence and description of *Alistipes senegalensis* sp. nov.

**DOI:** 10.4056/sigs.2625821

**Published:** 2012-07-20

**Authors:** Ajay Kumar Mishra, Gregory Gimenez, Jean-Christophe Lagier, Catherine Robert, Didier Raoult, Pierre-Edouard Fournier

**Affiliations:** 1 Unité de Recherche sur les Maladies Infectieuses et Tropicales Emergentes, Faculté de médecine, Aix-Marseille Université, Marseille, France

**Keywords:** *Alistipes senegalensis*, genome

## Abstract

*Alistipes senegalensis* strain JC50^T^ is the type strain of *A. senegalensis* sp. nov., a new species within the *Alistipes* genus. This strain, whose genome is described here, was isolated from the fecal flora of an asymptomatic patient. *A. senegalensis* is an anaerobic Gram-negative rod-shaped bacterium. Here we describe the features of this organism, together with the complete genome sequence and annotation. The 4,017,609 bp long genome (1 chromosome, but no plasmid) contains 3,113 protein-coding and 50 RNA genes, including 5 rRNA genes.

## Introduction

*Alistipes senegalensis* strain JC50^T^ (= CSUR P156= DSM 25460) is the type strain of *A. senegalensis* sp. nov. This bacterium was isolated from the stool of a healthy Senegalese patient as part of a “culturomics” study aiming at cultivating all species in human feces, individually.

Bacterial species definition is a matter of debate. This is notably due to the high cost, poor reproducibility and inter-laboratory comparability of the “gold standard” of DNA-DNA hybridization and G+C content determination [1]. In contrast, the development of PCR and sequencing methods, both of which are now widely available and cost-effective, has profoundly changed the way of classifying prokaryotes. Using 16S rRNA sequences with internationally-agreed upon cutoff values, despite variations among taxa, enabled the taxonomic classification or reclassification of hundreds of taxa [2]. More recently, high throughput genome sequencing and mass spectrometric analyses of bacteria have given unprecedented access to a wealth of genetic and proteomic information [3]. As a consequence, we propose to use a polyphasic approach [4] to describe new bacterial taxa that includes their genome sequence, MALDI-TOF spectrum and major phenotypic characteristics (habitat, Gram staining, culture and metabolic characteristics, and when applicable, pathogenicity). Here we present a summary classification and a set of features for *A. senegalensis* sp. nov. strain JC50^T^ together with the description of the complete genomic sequencing and annotation. These characteristics support the creation of the *A. senegalensis* species.

The genus *Alistipes* (Rautio *et al*. 2003) was created in 2003 [5] and is composed of strictly anaerobic Gram-negative rods that resemble the *Bacteroides fragilis* group in that most species are bile-resistant and indole-positive; however, they are only weakly saccharolytic and most species produce light brown pigment only on laked rabbit blood agar [6]. The genus *Alistipes* contains five species namely *A. finegoldii*, *A. putredinis* [5], *A. indistinctus* [7], *A. onderdonkii* and *A. shahii* [8].

The natural habitat of the genus *Alistipes* is unknown but most of the species have mostly been isolated from blood samples, appendiceal tissue samples, perirectal and brain abscess material [9,10]. Predisposing factors to *Alistipes sp.* bacteremia include malignant neoplasms, recent gastrointestinal or obstetric-gynecologic surgery, intestinal obstruction, and use of cytotoxic agents or corticosteroids [9]. A 16S rRNA phylogenetic analysis revealed that *A. senegalensis* is closely related to *A. shahii*. To the best of our knowledge, our report is the first to report the isolation of *Alistipes sp.* from the normal fecal flora.

### Classification and features

A stool sample was collected from a healthy 16-year-old male Senegalese volunteer patient living in Dielmo (rural villages in the Guinean-Sudanian zone in Senegal), who was included in a research protocol. The patient gave an informed and signed consent, and the agreement of the National Ethics Committee of Senegal and the local ethics committee of the IFR48 (Marseille, France) (agreement 09-022), were obtained. The fecal specimen was preserved at -80°C after collection and sent to Marseille. Strain JC50^T^ (Table 1) was isolated in March 2011 by anaerobic cultivation on Schaedler agar with kanamycin and vancomycin (Becton Dickinson, Heidelberg, Germany).

**Table 1:** Classification and general features of *Alistipes senegalensis* strain JC50^T^

**MIGS ID**	**Property**	**Term**	**Evidence code^a^**
		Domain *Bacteria*	TAS [11]
		Phylum *Bacteroidetes*	TAS [12,13]
		Class *Bacteroidia*	TAS [12,14]
	Current classification	Order *Bacteroidales*	TAS [12,15]
		Family *Rikenellaceae*	TAS [12,16]
		Genus *Alistipes*	TAS [12,17]
		Species *Alistipes senegalensis*	IDA
		Type strain JC50^T^	IDA
	Gram stain	negative	IDA
	Cell shape	bacilli	IDA
	Motility	nonmotile	IDA
	Sporulation	nonsporulating	IDA
	Temperature range	mesophileic	IDA
	Optimum temperature	37°C	IDA
MIGS-6.3	Salinity	growth in BHI medium + 1% NaCl	IDA
MIGS-22	Oxygen requirement	anaerobic	IDA
	Carbon source	unknown	
	Energy source	unknown	
MIGS-6	Habitat	human gut	IDA
MIGS-15	Biotic relationship	free living	IDA
MIGS-14	Pathogenicity	unknown	
	Biosafety level	2	
	Isolation	human feces	
MIGS-4	Geographic location	Senegal	IDA
MIGS-5	Sample collection time	September 2010	IDA
MIGS-4.1	Latitude	14.49740	IDA
MIGS-4.2	Longitude	-14.452362	IDA
MIGS-4.3	Depth	surface	IDA
MIGS-4.4	Altitude	51 m above sea level	IDA

Evidence codes - IDA: Inferred from Direct Assay; TAS: Traceable Author Statement (i.e., a direct report exists in the literature); NAS: Non-traceable Author Statement (i.e., not directly observed for the living, isolated sample, but based on a generally accepted property for the species, or anecdotal evidence). These evidence codes are from the Gene Ontology project [18]. If the evidence is IDA, then the property was directly observed for a live isolate by one of the authors or an expert mentioned in the acknowledgements.

**Figure 1:**
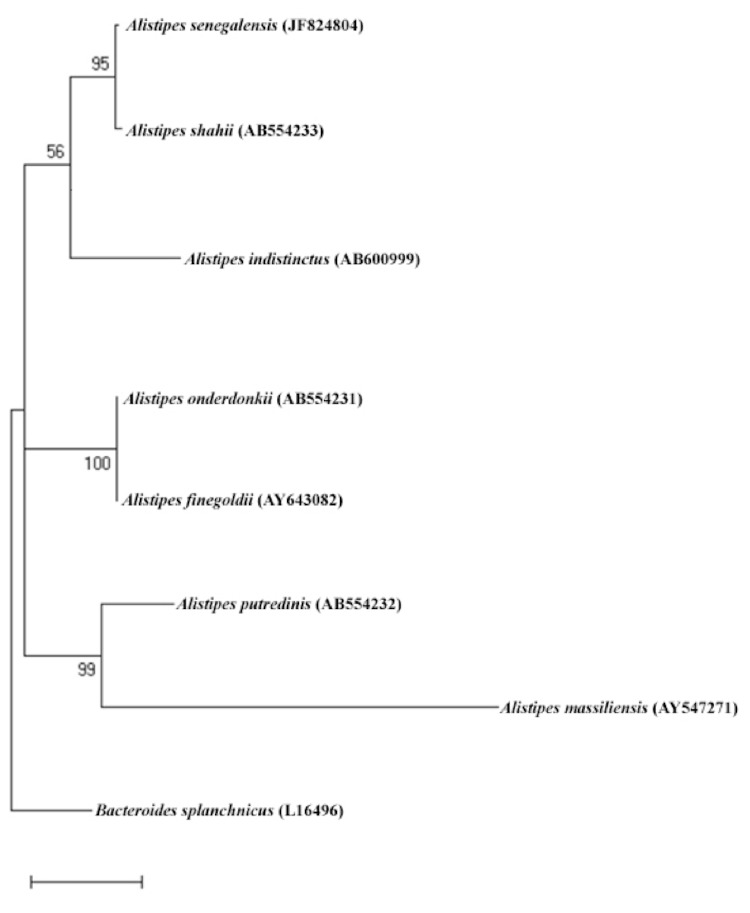
Phylogenetic tree highlighting the position of *Alistipes senegalensis* strain JC50^T^ relative to other type strains within the *Alistipes* genus. GenBank accession numbers are indicated in parentheses. The tree was inferred from the comparison of 16S rRNA gene sequence. Sequences were aligned using CLUSTALW, and phylogenetic inferences obtained using the maximum-likelihood method within the MEGA software. Numbers at the nodes are bootstrap values obtained by repeating 500 times the analysis to generate a majority consensus tree. *Bacteroides splanchnicus* was used as an outgroup. The scale bar represents a 2% nucleotide sequence divergence.

The strain exhibited 97.0%, 16S rRNA nucleotide sequence similarity with *A. shahii*, the phylogenetically-closest validly published *Alistipes* species.(Figure 1). Although the level of sequence similarity of the 16S rRNA gene sequence is not uniform across taxa, this value was lower than the 98.7% 16S rRNA gene sequence threshold recommended by Stackebrandt and Ebers to delineate a new species without carrying out DNA-DNA hybridization [19].

**Figure 2:**
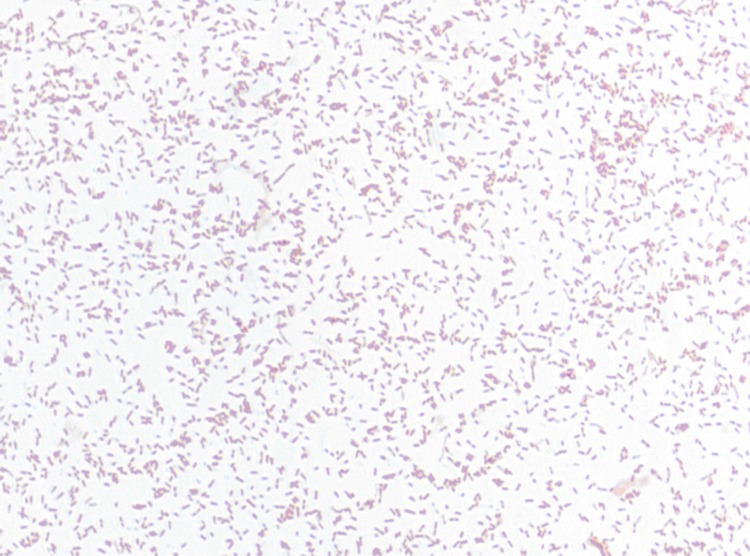
Gram staining of *A. senegalensis* strain JC50^T^

**Figure 3:**
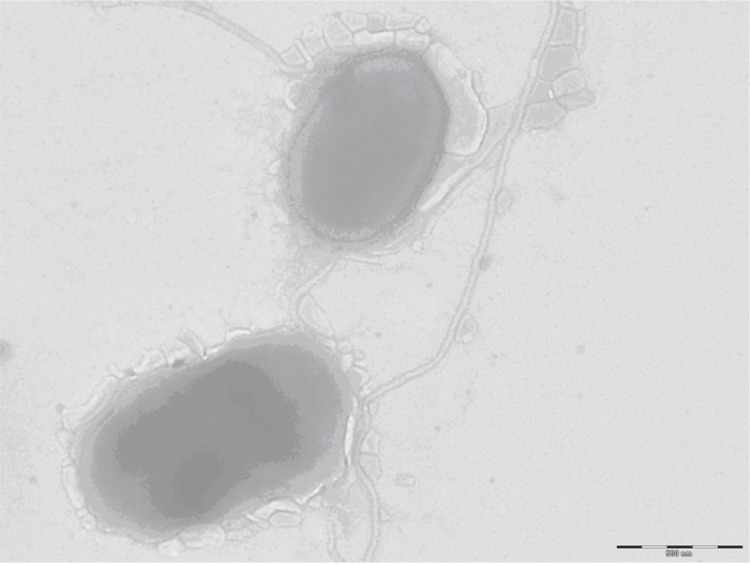
Transmission electron microscopy of *A. senegalensis* strain JC50^T^, using a Morgani 268D (Philips) at an operating voltage of 60kV.The scale bar represents 900 nm.

Different growth temperatures (25, 30, 37, 45°C) were tested; no growth occurred at 25°C and 45°C, growth occurred between 30 and 37°C, and optimal growth was observed at 37°C. Colonies were 0.2 to 0.3 mm in diameter on blood-enriched Columbia agar and Brain Heart Infusion (BHI) agar. Growth of the strain was tested under anaerobic and microaerophilic conditions using GENbag anaer and GENbag microaer systems, respectively (BioMérieux), and in the presence of air, with or without 5% CO_2_ and in aerobic conditions. The optimal growth of the strain was obtained anaerobically, with weak growth being observed under microaerophilic conditions, and no growth observed under aerobic conditions. Gram staining showed Gram negative bacilli. A motility test was negative. Cells grown on agar are Gram-negative rod-shaped bacteria (Figure 2) and have a mean diameter of 0.56 µm (Figure 3) by electron microscopy.

Strain JC50^T^ exhibited a catalase activity but no oxidase activity. Using API Rapid ID 32A, a positive reaction was obtained for mannose fermentation, proline arylimidase, leucyl glycine arylamidase, alanine arylamidase. A weak reaction was obtained for indole production, α-galactosidase, β-galactosidase, β-glucuronidase, arginine arlyamidase and glycine arylamidase. *A. senegalensis* is susceptible to penicillin G, imipeneme, amoxicillin + clavulanic acid and clindamycin but resistant to metronidazole and vancomycin.

**Figure 4:**
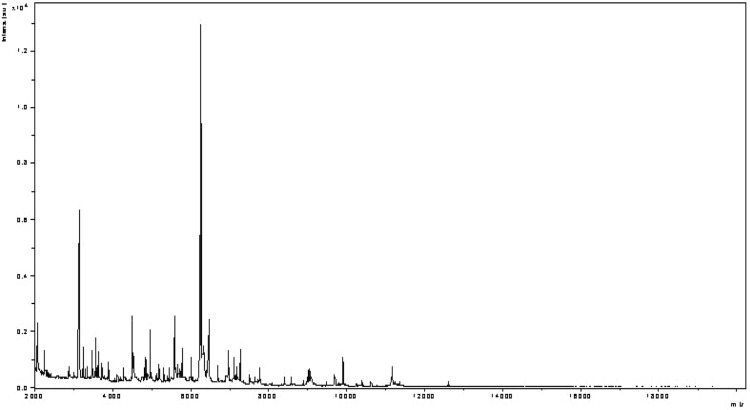
Reference mass spectrum from *A. senegalensis* strain JC50^T^. Spectra from 12 individual colonies were compared and a reference spectrum was generated.

Matrix-assisted laser-desorption/ionization time-of-flight (MALDI-TOF) MS protein analysis was carried out as previously described [20]. Briefly, a pipette tip was used to pick one isolated bacterial colony from a culture agar plate, and spread as a thin film on an MTP 384 MALDI-TOF target plate (Bruker Daltonics, Germany). Four distinct deposits were done for strain JC50^T^ from four isolated colonies. Each smear was overlaid with 2µL of matrix solution (saturated solution of alpha-cyano-4-hydroxycinnamic acid) in 50% acetonitrile, 2.5% tri-fluoracetic acid, and allowed to dry for five minutes. Measurements were performed with a Microflex spectrometer (Bruker). Spectra were recorded in the positive linear mode for the mass range of 2,000 to 20,000 Da (parameter settings: ion source 1 (ISI), 20kV; IS2, 18.5 kV; lens, 7 kV). A spectrum was obtained after 675 shots at a variable laser power. The time of acquisition was between 30 seconds and 1 minute per spot. The four JC50^T^ spectra were imported into the MALDI Bio Typer software (version 2.0, Bruker) and analyzed by standard pattern matching (with default parameter settings) against the main spectra of 2,843 bacteria, including spectra from three validly published *Alistipes* species used as reference data, in the Bio Typer database. The method of identification included the m/z from 3,000 to 15,000 Da. For every spectrum, 100 peaks at most were taken into account and compared with the spectra in database. A score enabled the presumptive identification, or discrimination, from the tested species: a score ≥ 2 with a validated species enabled the identification at the species level; a score ≥ 1.7 but < 2 enabled the identification at the genus level; and a score < 1.7 did not enable any identification. Spectra were compared with the Bruker database that contained spectra from the three validated *Alistipes* species. No significant score was obtained, thus suggesting that our isolate was not a member of a known species. We incremented our database with the spectrum from strain JC50^T^ (Figure 4).

## Genome sequencing and annotation

### Genome project history

The organism was selected for sequencing on the basis of its phylogenetic position and 16S rRNA similarity to other members of the *Alistipes* genus, and is part of a “culturomics” study of the human digestive flora aiming at isolating all bacterial species within human feces. It was the second genome of an *Alistipes* species and the first genome of *Alistipes senegalensis* sp. nov. A summary of the project information is shown in Table 2. The Genbank accession number is CAHI00000000 and consists of forty contigs. Table 2 shows the project information and its association with MIGS version 2.0 compliance [5].

**Table 2:** Project information

**MIGS ID**	**Property**	**Term**
MIGS-31	Finishing quality	High-quality draft
MIGS-28	Libraries used	One 454 paired end 3-kb library
MIGS-29	Sequencing platforms	454 GS FLX Titanium
MIGS-31.2	Fold coverage	35
MIGS-30	Assemblers	Newbler version 2.5.3
MIGS-32	Gene calling method	Prodigal
	INSDC ID GenBank ID	2000019201 CAHI00000000
	Genbank Date of Release	January 31, 2012
	Gold ID	Gi12116
	NCBI project ID	82331
MIGS-13	Project relevance	Study of the human gut microbiome

### Growth conditions and DNA isolation

*A. senegalensis* sp. nov. strain JC50^T^, CSUR P156, was grown on blood agar medium at 37°C. Twelve petri dishes were spread and resuspended in 6×100µl of G2 buffer (EZ1 DNA Tissue kit, Qiagen). A first mechanical lysis was performed by glass powder on the Fastprep-24 device (Sample Preparation system) from MP Biomedicals, USA during 2×20 seconds. DNA was then incubated for a lysozyme treatment (30 minutes at 37°C) and extracted through the BioRobot EZ 1 Advanced XL (Qiagen). The DNA was then concentrated and purified on a Qiamp kit (Qiagen). The yield and the concentration was measured by the Quant-it Picogreen kit (Invitrogen) on the Genios_Tecan fluorometer at 62.7 ng/µl.

### Genome sequencing and assembly

This project was loaded twice on a ¼ region for the paired end application and once on a ⅛ region for the shotgun on PTP Picotiterplates. The shotgun library was constructed with 500 ng of DNA as described by the manufacturer Roche with the GS Rapid library Prep kit. DNA (5µg) was mechanically fragmented on the Hydroshear device (Digilab, Holliston, MA, USA) with an enrichment size at 3-4kb. The DNA fragmentation was visualized through the Agilent 2100 BioAnalyzer on a DNA labchip 7500 with an optimal size of 3.563kb.The library was constructed according to the 454_Titanium paired end protocol and manufacturer. Circularization and nebulization were performed and generated a pattern with an optimal at 377 bp. After PCR amplification through 15 cycles followed by double size selection, the single stranded paired end library was then quantified on the Quant-it Ribogreen kit (Invitrogen) on the Genios_Tecan fluorometer at 215pg/µL. The library concentration equivalence was calculated as 10.5E+08 molecules/µL. The library was stocked at -20°C until use.

The shotgun library was clonally amplified with 3 cpb in 3emPCR reactions with the GS Titanium SV emPCR Kit (Lib-L) v2 leading to 13.93% yield of the emPCR. The paired end library was clonally amplified with 1cpb in 4 SV-emPCR reactions leading to 17.56% yield was in the range of 5 to 20% from the Roche procedure. 790,000 beads for a ¼ Region and 340,000 beads for a ⅛ region were loaded on the GS Titanium PicoTiterPlates PTP Kit 70×75 sequenced with the GS Titanium Sequencing Kit XLR70.

The runs were performed overnight and then analyzed on the cluster through the gsRunBrowser _Roche. Data from 78.55 Mb of passed filter wells were generated with an average of length of 228 bp for the paired end library, and 51.3 Mb with an average length of 417 bp were obtained from the shotgun library. The global passed filter sequences were assembled on the gsAssembler_Roche with 90% identity and 40bp as overlap. The final assembly into 4 scaffolds and 40 large contigs (>1500bp) generated a genome size of 4.01 Mb.

### Genome annotation

Open Reading Frames (ORFs) were predicted using Prodigal [21] with default parameters but the predicted ORFs were excluded if they were spanning a sequencing GAP region. The predicted bacterial protein sequences were searched against the GenBank database [22] and the Clusters of Orthologous Groups (COG) databases using BLASTP. The tRNAScanSE tool [23] was used to find tRNA genes, whereas ribosomal RNAs were found by using RNAmmer [24] and BLASTn against the NR database. ORFans were identified if their BLASTP *E*-value were lower than 1e-03 for alignment length greater than 80 amino acids. If alignment lengths were smaller than 80 amino acids, we used an *E*-value of 1e-05. Such parameter thresholds have already been used in previous works to define ORFans. To estimate the mean level of nucleotide sequence similarity at the genome level between *Alistipes* species, we compared the ORFs only using BLASTN and the following parameters: a query coverage of ≥ 70% and a minimum nucleotide length of 100 bp.

## Genome properties

**Figure 5:**
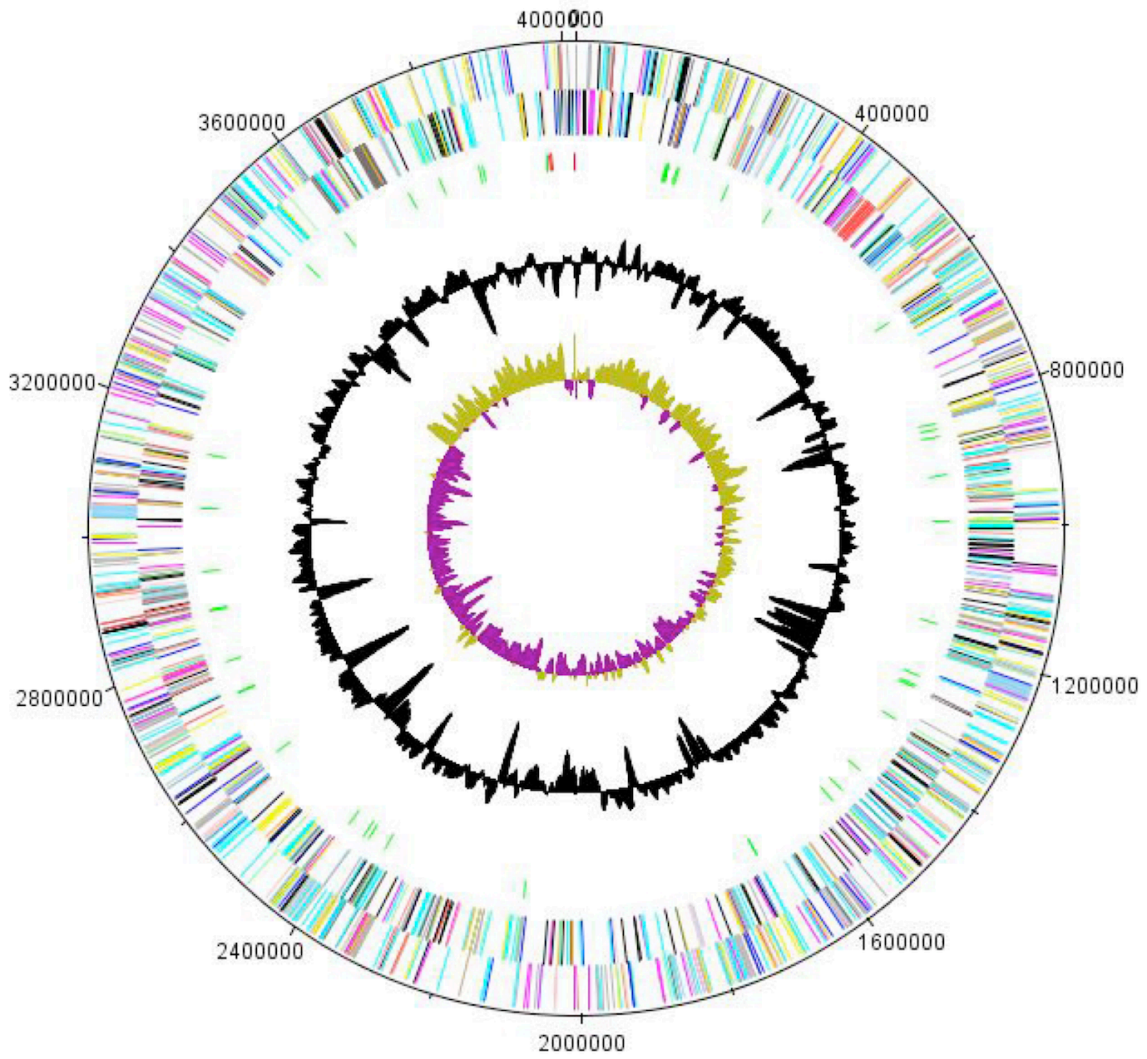
Graphical circular map of the chromosome. From outside to the center: Genes on the forward strand (colored by COG categories), genes on the reverse strand (colored by COG categories), RNA genes (tRNAs green, rRNAs red), GC content, and GC skew.

The genome is 4,017,609 bp long (1 chromosome but no plasmid) with a 58.40% GC content (Figure 5 and Table 3). Of the 3,163 predicted genes, 3,113 were protein-coding genes, and 50 were RNAs. A total of 1,977 genes (62.50%) were assigned a putative function. Eighty-one genes were identified as ORFans (2.6%). The remaining genes were annotated as hypothetical proteins. The properties and statistics of the genome are summarized in Tables 3 and distribution of genes into COG functional categories is presented in Table 4. 

**Table 3:** Nucleotide content and gene count levels of the genome

**Attribute**	**Value**	**% of total^a^**
Genome size (bp)	4,017,609	
DNA coding region (bp)	3,670,587	91.36
DNA G+C content (bp)	2,3462,84	58.40
Total genes	3,163	100
RNA genes	50	1.58
Protein-coding genes	3,113	98.41
Genes with function prediction	1,977	62.50
Genes assigned to COGs	1,863	58.90
Genes with peptide signals	712	22.51
Genes with transmembrane helices	645	20.39

^a^ The total is based on either the size of the genome in base pairs or the total number of protein coding genes in the annotated genome

**Table 4:** Number of genes associated with the 25 general COG functional categories

**Code**	**Value**	**%age**^a^	**Description**
J	140	4.49	Translation
A	0	0	RNA processing and modification
K	149	4.78	Transcription
L	136	4.37	Replication, recombination and repair
B	0	0	Chromatin structure and dynamics
D	18	0.58	Cell cycle control, mitosis and meiosis
Y	0	0	Nuclear structure
V	44	1.41	Defense mechanisms
T	89	2.85	Signal transduction mechanisms
M	166	5.33	Cell wall/membrane biogenesis
N	7	0.22	Cell motility
Z	0	0	Cytoskeleton
W	0	0	Extracellular structures
U	41	1.32	Intracellular trafficking and secretion
O	67	2.15	Posttranslational modification, protein turnover, chaperones
C	134	4.30	Energy production and conversion
G	222	7.13	Carbohydrate transport and metabolism
E	155	4.98	Amino acid transport and metabolism
F	54	1.73	Nucleotide transport and metabolism
H	73	2.34	Coenzyme transport and metabolism
I	45	1.45	Lipid transport and metabolism
P	138	4.43	Inorganic ion transport and metabolism
Q	18	0.58	Secondary metabolites biosynthesis, transport and catabolism
R	281	9.03	General function prediction only
S	112	3.60	Function unknown
-	1,250	32.89	Not in COGs

^a^ The total is based on the total number of protein coding genes in the annotated genome.

### Comparison with the genomes from other *Alistipes* species

To date, the genome from *Alistipes shahii* strain WAL 8301 is the only genome from the *Alistipes* genus that has been sequenced. By comparison with *A. shahaii*, *A. senegalensis* exhibited a higher G+C content (57.2% *vs* 58.40%, respectively), a higher number of genes (2,616 *vs* 3,163) and a smaller number of genes with peptide signal (989 *vs* 712). Moreover, *A. senegalensis* had higher ratios of genes per Mb (696 *vs* 788) and a comparable number of genes assigned to COGs (58.9 vs 59.3). However, the distribution of genes into COG categories (Table 4) was highly similar in both genomes. In addition, *A. senegalensis* and *A. shahaii* shared a mean 89.9% (range 78.4-100%) sequence similarity at the genome level.

## Conclusion

On the basis of phenotypic, phylogenetic and genomic analyses, we formally propose the creation of *Alistipes senegalensis* sp. nov. that contains the strain JC50^T^. This bacterium has been found in Senegal.

### Description of *Alistipes senegalensis* sp. nov.

*Alistipes senegalensis* (se.ne.gal.e’n.sis. L. gen. masc. n. *senegalensis*, of Senegal, the country of origin of *Alistipes senegalensis*).

Colonies are 0.2 to 0.3 mm in diameter on blood-enriched Columbia agar and Brain Heart Infusion (BHI) agar. Cells are rod-shaped with a mean diameter of 0.56 µm. Optimal growth is achieved anaerobically. Weak growth is observed in microaerophilic conditions. No growth is observed in aerobic conditions. Growth occurred between 30-37°C, with optimal growth observed at 37°C, in BHI medium + 5% NaCl. Cells stain Gram negative and are non-motile. Catalase, α-galactosidase, β-galactosidase, β-glucuronidase, arginine arlyamidase, glycine arylamidase, proline arylimidase, leucyl glycine arylamidase, and alanine arylamidase activities are present. Mannose fermentation and indole production are also present. Oxidase activity is absent. Cells are susceptible to penicillin G, amoxicillin + clavulanic acid, imipeneme and clindamycin but resistant to metronidazole. The G+C content of the genome is 58.40%. The 16S rRNA and genome sequence are deposited in GenBank under accession numbers JF824804 and CAHI00000000, respectively.

The type strain JC50^T^ (= CSUR P156 = DSM 25460) was isolated from the fecal flora of a healthy patient in Senegal. 
